# Impact of weather seasonality and sexual transmission on the spread of Zika fever

**DOI:** 10.1038/s41598-019-53062-z

**Published:** 2019-11-19

**Authors:** Attila Dénes, Mahmoud A. Ibrahim, Lillian Oluoch, Miklós Tekeli, Tamás Tekeli

**Affiliations:** 1 Bolyai Institute, University of Szeged, Aradi vértanúk tere 1., Szeged, H-6720 Hungary; 2 Department of Mathematics, Faculty of Science, Mansoura University, Mansoura, 35516 Egypt

**Keywords:** Infectious diseases, Viral infection

## Abstract

We establish a compartmental model to study the transmission of Zika virus disease including spread through sexual contacts and the role of asymptomatic carriers. To incorporate the impact of the seasonality of weather on the spread of Zika, we apply a nonautonomous model with time-dependent mosquito birth rate and biting rate, which allows us to explain the differing outcome of the epidemic in different countries of South America: using Latin Hypercube Sampling for fitting, we were able to reproduce the different outcomes of the disease in various countries. Sensitivity analysis shows that, although the most important factors in Zika transmission are the birth rate of mosquitoes and the transmission rate from mosquitoes to humans, spread through sexual contacts also highly contributes to the transmission of Zika virus: our study suggests that the practice of safe sex among those who have possibly contracted the disease, can significantly reduce the number of Zika cases.

## Introduction

## Zika virus

**Figure 1:**
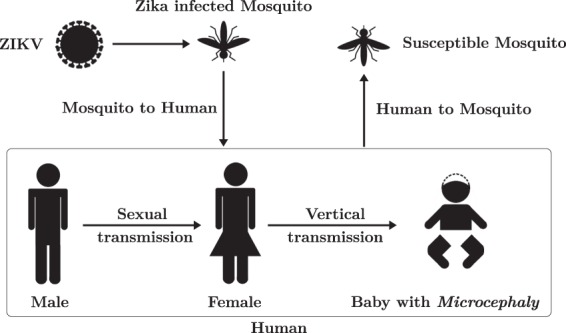
Biology of Zika Virus (ZIKV). The figure shows modes of transmission and illustrates the critical pathological manifestation (microcephaly) associated with Zika infection.

Zika virus (ZIKV) is a virus belonging to the family *Flaviviridae*, primarily transmitted to humans by the bites of infected female mosquitoes from the *Aedes* genus, such as *Aedes aegypti* and *Aedes albopictus*^1^, widespread in tropical and subtropical regions and spreading in temperate areas as well. ZIKV is related to other arboviruses like chikungunya and dengue. Beside the major source of transmission (mosquito bites) the virus can also be passed on through other means. Unlike the above-listed diseases, Zika can be transmitted through sexual contacts, mostly from men to women^2^. The disease can be passed from a person with Zika before the start of the symptoms, while having symptoms, and after their symptoms end. The virus may also be passed by asymptomatic carriers^3^. Studies suggest that ZIKV can remain in semen longer (possibly even six months) than in other body fluids^4^. Another important way of transmission is from expectant mother to her child during pregnancy or around the time of birth. This may lead to Congenital Zika Syndrome the symptoms of which include microcephaly and a specific pattern of brain damage. The virus can also be transmitted through blood transfusion and breastfeeding^5,6^. Figure 1 shows the possible methods of Zika transmission.

The infection, known as Zika fever or Zika virus disease, shares clinical signs and symptoms with dengue and chikungunya fever, including mild fever, rash, conjunctivitis and joint pain. It has also been linked to severe neurological diseases, such as Guillain–Barré syndrome (a muscle weakness caused by the immune system damaging the peripheral nervous system)^7^ and microcephaly, a medical condition in which the brain does not develop properly resulting in a head smaller than its normal size. ZIKV poses a serious threat to public health internationally due to the continuous geographic expansion of both the virus and its mosquito vectors^8^. As for today, there are no known vaccines or specific therapies available for treatment and prevention^9^.

**Figure 2:**
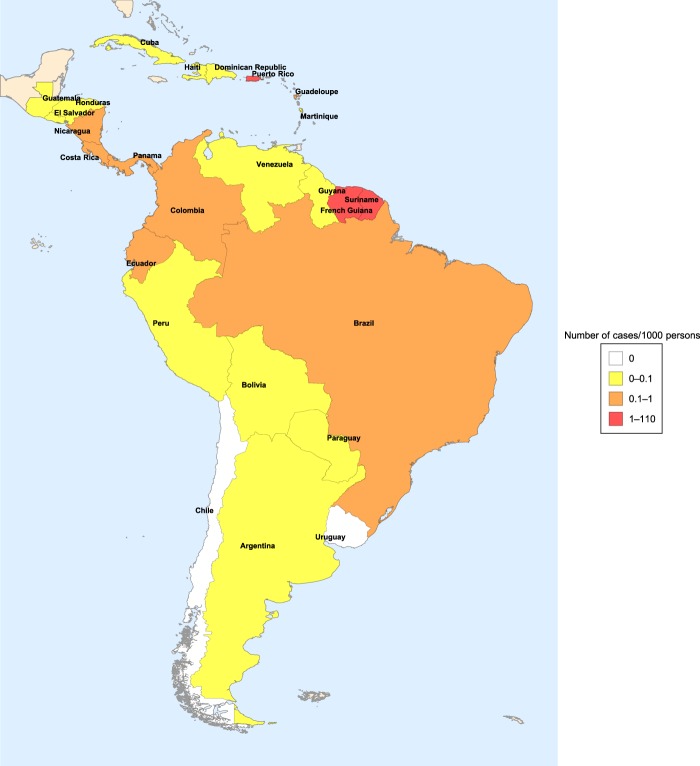
Incidence of Zika fever in Central and South American countries affected by the epidemic 2015–2017^16^.

First identified in 1947 in a rhesus monkey in the Zika forest in Uganda^10^, the virus was recovered in 1948 from the mosquito *Aedes africanus*, caught in the Zika forest^11^. The first human cases were reported in Uganda and Tanzania in 1952^12^. The first large outbreak in humans took place in 2007 in the Yap Island and later in French Polynesia, Easter Island, the Cook Islands, New Caledonia. In 2008, a scientist contracted Zika fever in Senegal and after returning to the US, he infected his wife. This is the first documented case of sexual transmission of a disease transmitted by insects^13^. The first cases in South America were discovered in Brazil in the spring of 2015, and several other countries from the region confirmed Zika cases at the end of 2015 and early 2016 (for the incidence of Zika fever in Central and South American countries affected by the epidemic 2015–2017, see Fig. 2). The rapid spread of Zika in Brazil can be attributed to the completely susceptible population, high population density, tropical climate and inadequate control of *Aedes* mosquitoes^14,15^. In October 2015, Brazil reported an increase in the number of microcephaly cases among newborns. In November, Zika virus genome was detected in the blood and tissues of a baby born with microcephaly in Brazil. In January 2016, intrauterine transmission of Zika virus was detected for the first time in two pregnant women in Brazil whose fetuses were diagnosed with microcephaly. An increased number of cases of Guillain–Barré syndrome was also reported from other countries of South America.

The course of the Zika epidemic was different in various countries of South America. The reason behind this is most probably that these countries are very heterogeneous in their climatic, geographic, demographic characteristics. Basically, we can distinguish two different situations. In one part of the countries, e.g. Colombia, Puerto Rico, Suriname, there was a single outbreak, while in other countries, including Bolivia, Costa Rica, Ecuador, one can observe two major peaks in two successive years^16^.

Although the number of Zika cases has declined since the virus was first introduced in the Americas, in February 2018, Zika fever was included in WHO’s Blueprint list of priority diseases to be prioritized for research and development^17^. Temperature is known to be a strong driver of vector-borne disease transmission^18^, hence, considering climate change, a probable extension of the distribution of carrying mosquitoes implying a possible introduction of Zika into so far unaffected regions, Zika virus will most probably continue to be an important menace in the future.

## Mathematical models for Zika transmission

Various mathematical models have been established to study the transmission dynamics of the spread of Zika virus. Gao *et al*.^19^ introduced a compartmental model of Zika spread considering vector-borne and sexual transmission proposing an SEIR-type model for the human population and an SEI-chain for vectors. They separated asymptomatically infected humans from those who had symptoms, but males and females were not differentiated. The authors used historical data to approximate the parameters of the system. Baca-Carrasco *et al*.^20^ proposed compartmental models considering vector-borne and sexual transmission (with the two sexes differentiated) and migration as well, showing that sexual transmission influences the magnitude of the outbreaks. Suparit *et al*.^21^ studied the spread of Zika fever in Bahia, Brazil considering two vector control strategies: reducing mosquito biting rates and mosquito population size. The model also includes the influences of seasonal change on the ZIKV transmission dynamics via time-varying mosquito biting rate, however, it does not take into account human-to-human transmission. There are also papers which consider the importance of weather and climate changes in the models, see e.g. Caminade *et al*.^14^ and Mordecai *et al*.^22^. Guzzetta *et al*.^23^, Rocklöv *et al*.^24^, Marini *et al*.^25^ studied models for the spread of Zika to new areas. Further models for Zika transmission are studied e.g. in^26–33^.

The majority of models so far did not consider the seasonality of the spread of the disease, induced by the seasonality of mosquito population size. In the present work, we establish a compartmental model for Zika transmission, considering the important features of the disease included in earlier models such as mosquito-borne and sexual transmission, asymptomatically infected people contributing to the spread of the disease, a prolonged infectivity through sexual transmission, and we also incorporate the effect of the seasonality of weather by introducing time-dependent (periodic) parameters. The resulting model is able to reproduce the different outcomes of the epidemic in various countries affected by Zika, and enables us to obtain a better understanding of the role of different parameters.

## Methods

### Compartmental model with time-dependent parameters

As described in the Introduction, the Zika epidemic had different outcome in the countries of South America. Earlier mathematical models, though able to provide a good fit for the one-peak case, were unable to reproduce the situation with two peaks^19,20^. The reason for this is that these models did not consider the annual change of weather conditions and the consequent annual fluctuation of the size of mosquito populations. This motivated us to establish a new compartmental model for Zika virus transmission which also considers the periodicity of weather. Furthermore, to assure that our model properly describes the real world situation, we also considered both symptomatic and asymptomatic carriers of the disease, and the two sexes were differentiated to make the model applicable for evaluation of the role of sexual transmission of Zika as well. As the number of sexual transmissions from women to men is reported to be very small compared to transmission in the other direction, in this model we only consider sexual transmission from men to women^34,35^.

**Figure 3:**
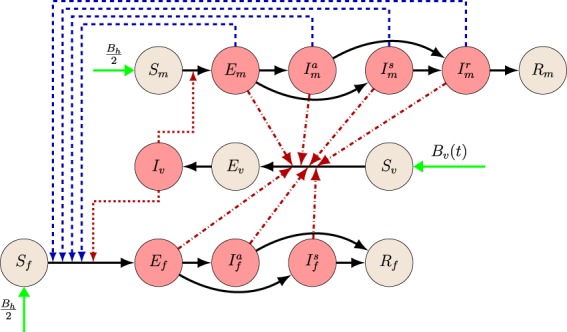
Dynamics of Zika virus spread considering two sexes and involving vectorial and sexual transmission. Male and female human and vector populations are differentiated by the subscripts $m$, $f$, $v$, respectively. Red nodes are infectious and brown nodes are non-infectious. Black solid arrows show the progression of infection. Blue dashed arrows show male-to-female human transmission. Red dash-dotted lines show human-to-mosquito transmission and red dotted lines show mosquito-to-human transmission. Green arrows show natural birth.

To incorporate all of the above features in our study, our model includes 14 compartments. Male human, female human and vector compartments are differentiated by the subscripts $m,f,v$, respectively. Susceptible humans (${S}_{m}$ and ${S}_{f}$) are those who can be infected by Zika virus. After contracting the disease, one moves to the exposed class (${E}_{m},{E}_{f}$), these individuals do not have any symptoms yet. After the incubation period, one moves either to the symptomatically infected class (${I}_{m}^{s},{I}_{f}^{s}$) or to the asymptomatically infected class (${I}_{m}^{a},{I}_{f}^{a}$), depending on whether that individual develops the symptoms or not. Symptomatically and asymptomatically infected women move to the recovered compartment ${R}_{f}$ after recovery, however, for men, there is an additional convalescent compartment (${I}_{m}^{r}$) for those who have recovered from the disease but can still transmit it through sexual contact. After the convalescent phase, men move to the recovered class ${R}_{m}$. We emphasize that the infectious classes $E,{I}^{s},{I}^{a},{I}^{r}$ are also distinguished by their differing transmission and recovery rates. For the mosquitoes, we have three compartments: susceptibles (${S}_{v}$), exposed (${E}_{v}$) and infected $({I}_{v})$. The transmission diagram of the model is shown in Fig. 3. The governing differential equations are specified in the Supplementary Information S.1, while the parameters applied in our work are described in Table 1.

**Table 1:** Description of parameters and fitted values in the case of Costa Rica and Suriname with ranges applied in the LHS sampling. Parameters where no range is given, are directly available (${B}_{h},d$) or determined by the time and size of peaks ($a,b$).

Parameter	Description	Range	Value (CRC)	Value (SUR)	Source
${B}_{h}$	Natural birth rate of humans (days${}^{-1}$)	–	171.073	21.619	^49^
$d$	Natural death rate of humans (days${}^{-1}$)	–	0.000034	0.000038	^49^
$\beta$	Transmission rate from infected humans to susceptible humans (days${}^{-1}$)	0.01–0.1	0.041 (0.041–0.057)	0.053 (0.047–0.057)	^19^
${\alpha }_{h}$	Baseline value of transmission rate from mosquitoes to humans (days${}^{-1}$)	0.03–0.75	0.141 (0.127–0.174)	0.142 (0.126–0.156)	^50,51^
${\alpha }_{v}$	Baseline value of transmission rate from humans to mosquitoes (days${}^{-1}$)	0.09–0.75	0.517 (0.468–0.577)	0.3 (0.235–0.319)	^50,51^
$\theta$	Proportion of asymptomatic infections	0.75–0.9	0.841 (0.841–0.853)	0.857 (0.824–0.86)	^19,52^
${\kappa }_{e}$	Relative human-to-human transmissibility of exposed to symptomatic humans	0.2–0.9	0.75 (0.737–0.762)	0.848 (0.842–0.855)	^19^
${\kappa }_{a}$	Relative human-to-human transmissibility of asymptomatic to symptomatic humans	0.2–0.8	0.491 (0.468–0.491)	0.306 (0.306–0.371)	^19^
${\kappa }_{r}$	Relative human-to-human transmissibility of convalescent to symptomatic humans	0.2–0.8	0.451 (0.414–0.475)	0.363 (0.338–0.382)	^19^
${\eta }_{e}$	Relative human-to-mosquito transmissibility of exposed to symptomatic humans	0.2–0.7	0.429 (0.281–0.429)	0.465 (0.421–0.494)	^19^
${\eta }_{a}$	Relative human-to-mosquito transmissibility of asymptomatic to symptomatic humans	0.2–0.7	0.516 (0.42–0.537)	0.586 (0.471–0.592)	^19^
${\gamma }_{a}$	Recovery rate of asymptomatically infected humans (days${}^{-1}$)	0.05–0.4	0.265 (0.256–0.279)	0.279 (0.276–0.293)	^19^
${\gamma }_{s}$	Recovery rate of symptomatically infected humans (days${}^{-1}$)	0.2–0.5	0.395 (0.365–0.429)	0.414 (0.405–0.427)	^53^
${\gamma }_{r}$	Recovery rate of convalescent humans (days${}^{-1}$)	0.01–0.07	0.041 (0.039–0.042)	0.062 (0.058–0.063)	^54,55^
${\nu }_{h}$	Human incubation rate (days${}^{-1}$)	0.1–0.5	0.191 (0.176–0.318)	0.391 (0.391–0.458)	^53^
${B}_{v}$	Baseline value of mosquito birth rate (days${}^{-1}$)	500–10000	9910	6699	Fitted
${\nu }_{v}$	Incubation rate in mosquitoes (days${}^{-1}$)	0.08–0.125	0.099 (0.096–0.105)	0.105 (0.102–0.106)	^51,56^
$1/\mu$	Mosquito life span (days)	7–21	7.9 (7.69–8.07)	7.66 (7.66–7.81)	^51^
$a$	Seasonality parameter	–	2.296	1.435	–
$b$	Seasonality parameter	–	269.221	194.743	–

### Parameter estimation and sensitivity

To study the phenomena described above, we fitted our model to data from South American countries with different outcomes of the epidemic. As examples for the cases with one or two peaks, we chose Suriname and Costa Rica respectively. To estimate the parameters which provide the best fit, we applied Latin Hypercube Sampling, which is a sampling method used in statistics to measure simultaneous variation of several parameter values (see, e.g.^36^). The method consists in generating a representative sample set from the parameter ranges for all fitted parameters shown in Table 1: to obtain a representative sample set of size $m$, all parameter ranges are divided into $m$ equal parts select one point in each subinterval. After obtaining the $m$ lists of samples, we combine them randomly, into $m$-tuples. Then, for all elements of this representative sample set, one numerically calculates the solutions of the model with the given parameter values. Finally, the least squares method is applied to obtain the parameters which give the best fit. However, it is important to note that due to the large number of parameters and the broad intervals of possible values of these, one cannot expect to find a single parameter set which perfectly fits the data of the epidemic, but rather to give a good approximation of the real situation and determine a region for each of the parameters so that the real values of the parameters fall in these intervals with a high probability. Hence, following the procedure described e.g. in^37^, we applied several rounds of LHS samplings as follows. In each round, we select the ten parameter sets which offer the best fits and the intervals for all parameters are narrowed down to the set between the minimal and maximal values for the given parameter among the ten best fitting parameter sets. Then, the next LHS sampling will take values from these narrower intervals. After performing this several times (eight times in our case), we obtain a reasonably small neighbourhood around the best fitting parameters. Apart from giving a good approximation of the parameter values, the fitting also allows us to estimate the burden of disease associated to sexual transmission, a novel phenomenon for mosquito-borne diseases, as well as to estimate the effect of the asymmetry of sexual transmission rate on the number of cases in the two sexes.

Using Partial Rank Correlation Coefficients analysis (PRCC^38^), we performed sensitivity analysis, to determine which of the parameters in our model have the most important effect on the transmission dynamics. As a response function for these simulations, we chose the cumulative number of new symptomatically infected cases. The sensitivity analysis based on the PRCC ranks the effect of the parameters on the response function (or outcome), while varying the parameters in their given ranges (parameters with higher positive (negative) PRCC values are positively (negatively) correlated with the response function).

### Basic reproduction number and instantaneous reproduction number

The estimation of the basic reproduction number (measuring the expected number of secondary infections generated by a single infected individual introduced into a completely susceptible population during his/her infection) is usually an utmost important question in the study of mathematical models of infectious diseases. The instantaneous reproduction number is considered when part of the population is immune. In mathematical models with periodic coefficients, the basic reproduction number can be obtained as the spectral radius of a linear integral operator on a space of periodic functions (for details, see^39^). In general, the actual value of the basic reproduction number cannot be calculated analytically, however, there exist methods to numerical approximate it (see Supplementary Information S.3 or e.g.^40^ for details). Besides determining the basic reproduction number of the periodic model, computing the formula for the basic reproduction number of the time-constant model obtained from the periodic model by setting the time-dependent parameters (mosquito birth rate and transmission rates between humans and mosquitoes) to constant also provides interesting results. The derivation of the formula for the basic reproduction number can be found in the Supplementary Information S.2. This formula gives us the basic reproduction number in any given time instant by substituting the parameter values into it, including the value of the time-dependent parameters at that given moment. This also allows us to calculate the instantaneous reproduction rate ${{\mathscr{R}}}_{{\rm{i}}{\rm{n}}{\rm{s}}{\rm{t}}}$ which estimates the average number of secondary cases per infectious case in a population made up of both susceptible and non-susceptible individuals and which can be obtained by multiplying the basic reproductive rate by the size of the susceptible fraction of the host population.

## Results

### Parameter estimation for countries with different outcome of the epidemic

Using the method described in Subsection 2.2, we fitted our model to data in countries where the epidemic outbreak had different outcome: Suriname, where there was a single peak of the epidemic and Costa Rica, where there were two peaks in two subsequent years.

**Figure 4:**
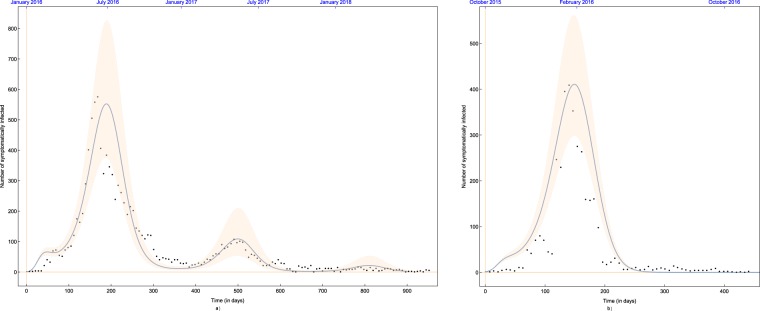
The best fitting solution with parameter values in Table 1 plotted with 99% confidence interval for (**a**) Costa Rica and (**b**) Suriname.

Figure 4 shows our model fitted to data from Suriname, in which country there was only one peak of the Zika epidemic, as well as to data from Costa Rica^16,41^. The best fitting solutions obtained with parameters given in Table 1 are depicted together with the 99% confidence range, which was obtained by letting for all parameters a 1% relative error w.r.t. the best fitting parameters. Our model gives a reasonably good fit, reproducing the two larger outbreaks of 2016 and 2017 and a modest number of cases in 2018. We note that the two-peak case could not have been reproduced using a time-constant model.

The results show that our model is able to reproduce both typical types of scenarios of the Zika epidemic. Depending on the parameter values characteristic of the given country, the simulations show that after one or more years, the number of susceptibles drops to a level where no further outbreak is possible, regardless of the annual periodicity of the number of mosquitoes caused by periodically changing weather conditions. The amount of newborns is not sufficient to provide a level of susceptibles which is enough to start a new outbreak.

The reasonably good fits obtained demonstrates that our model is able to reproduce both typical outcomes of the Zika epidemic, showing that the periodic change of weather, resulting in a setback in the number of new infections in autumn/winter, may also cause a recurrence of the epidemic, depending on the parameters characteristic for the given region, while under different circumstances, even the return of the warm, rainy season is insufficient to induce a new outbreak.

### Basic reproduction number and instantaneous reproduction number

We calculated numerically the basic reproduction number as the spectral radius of a linear integral operator (for details of the method, see e.g.^40^). This quantity serves as a threshold parameter for the eventual persistence of extinction of the epidemic. In the case of Costa Rica, we obtained the value ${{\mathscr{R}}}_{0}\approx 0.924$, while in the case of Suriname ${{\mathscr{R}}}_{0}\approx 0.737$. Both values are less than $1$, in accordance with the fact that eventually, in both countries, the epidemic died out. It is also important to note that, as both values are rather close to $1$, a sufficient increase of the appropriate parameters might lead to ${{\mathscr{R}}}_{0}$ becoming larger than $1$, which corresponds to the disease becoming endemic with an annual reappearance. Climate change or emergence of the disease in previously unaffected regions might lead to such alteration of the parameters, however, the details of predicting such a phenomenon is beyond the scope of the present study.

We also determined the formula for the basic reproduction number of the time-constant model obtained by setting the periodic parameters to constant (see details in Supplementary Information S.2).

**Figure 5:**
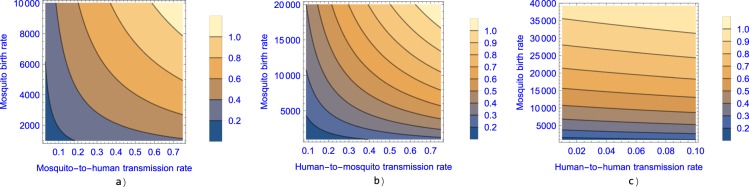
The contour plot of the basic reproduction number as a function of the mosquito birth rate (${B}_{v}$) and in (**a**) mosquito-to-human transmission rate (${\alpha }_{h}$), (**b**) human-to-mosquito transmission rate (${\alpha }_{v}$) and (**c**) human-to-human transmission rate ($\beta$).

In Fig. 5, we plot the basic reproduction number of the constant model as a function of mosquito birth rate, and human–mosquito transmission rates and human-to-human transmission rate (with the rest of the parameters set as obtained in the fitting to data from Suriname). The figure suggests that mosquito control and sexual protection are both important factors in the spread of Zika fever and that vector control might be insufficient to control the disease if sexual transmission rate is high.

**Figure 6:**
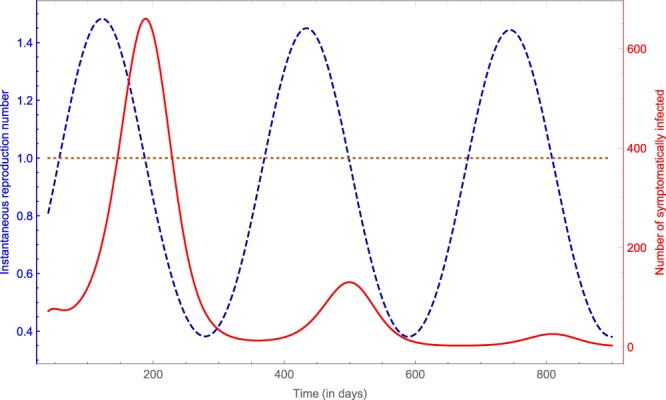
The instantaneous reproduction number (blue dashed line) and the number of symptomatically infected (red solid line) in Costa Rica, 2016–18.

Figure 6 shows the change of the instantaneous reproduction number together with the number of symptomatically infected people in Costa Rica, 2016–18. One can see that in each outbreak, the number of infected people starts to decrease, once the instantaneous reproduction number drops below 1. The maximal value of the instantaneous reproduction number is estimated to be around ${{\mathscr{R}}}_{{\rm{i}}{\rm{n}}{\rm{s}}{\rm{t}}}\approx 1.47$, while in Suriname it is estimated to be around ${{\mathscr{R}}}_{{\rm{i}}{\rm{n}}{\rm{s}}{\rm{t}}}\approx 1.45$. These values can be compared with earlier calculations of the basic reproduction number. Gao *et al*.^19^, using a compartmental model for data from Brazil, Colombia, and El Salvador estimated ${{\mathscr{R}}}_{0}=2.055$, Saad-Roy *et al*.^42^ estimated ${{\mathscr{R}}}_{0}=1.4$ for Brazil, which are close to our results. We note however, that some other studies estimated a higher value for the basic reproduction number: Towers *et al*.^43^, through an analysis of the exponential rise in clinically identified ZIKV cases gave an estimate of ${{\mathscr{R}}}_{0}=3.8$ for Barranquilla, Colombia, while Shutt *et al*.^44^ estimated the value of ${{\mathscr{R}}}_{0}$ to be between 4 and 6 in El Salvador and Suriname using a simple compartmental model.

### Difference in prevalence among women and men due to asymmetric sexual transmission

**Figure 7:**
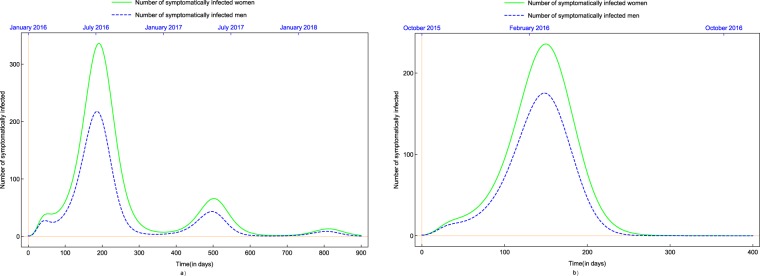
Number of symptomatically infected men and women in (**a**) Costa Rica and (**b**) Suriname.

As the main concern about the spread of Zika virus disease is mother-to-child transmission resulting in brain malformations, it is of primary importance to study the effect of the virus on women and estimate the number of cases among them. Moreover, several studies and news articles reported a higher number of Zika virus infections in women compared to cases in men^45–47^. It is probable that the higher male-to-female sexual transmission rate is a contributing factor to this skewing of the burden of disease toward women. Using our model, we compare the number of symptomatic cases in women and men (see Fig. 7). Based on our model, we estimate a 39% surplus in the cumulative number of symptomatically infected women in comparison with the cumulative number of symptomatically infected men in Suriname, while the same surplus is estimated 65% in Costa Rica. Lozier *et al*.^45^ reported a 62.5% surplus in Puerto Rico and 75% in Brazil and El Salvador, Cruz *et al*.^33^ estimated a 60% surplus in Rio de Janeiro, while Coelho *et al*.^46^ reported a 90% surplus, also in Rio de Janeiro. In comparison with these studies, our simulation shows similar or somewhat smaller surplus of Zika cases in women, though, the high number given in these studies might also be attributed to the fact that women (and especially pregnant women) suspected to have Zika are more likely to visit their doctors because of the potential risk of birth defects and potentially because women visit doctors more often than men, as hypothesized in^46^.

**Figure 8:**
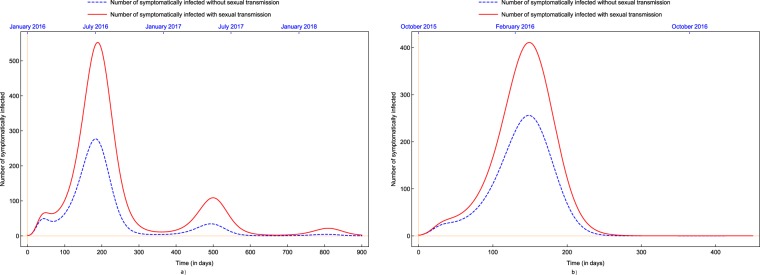
Number of symptomatically infected and estimated number of symptomatically infected in the absence of sexual transmission in (**a**) Costa Rica and (**b**) Suriname.

We also applied our model to estimate the effect of the presence of sexual transmission on the number of infected people. Figure 8 shows the actual number of symptomatically infected as well as the estimated number of symptomatically infected in the complete absence of sexual transmission. The estimation suggests that sexual transmission, a phenomenon earlier unknown in mosquito-borne diseases, significantly contributed to the number of Zika cases. More precisely, based on our model, we estimate that 32% of the total number of cases in Suriname and 54% in Costa Rica could be attributed to sexual transmission. Similar estimations were given in other studies as well, but a high level of uncertainty can be noticed^48^. Cruz *et al*.^33^ estimated that sexual transmission is responsible for 23% to 46% of the increment in the basic reproduction number. Towers *et al*.^43^ found that the fraction of cases due to sexual transmission was 0.23 $[0.01,0.47]$ with 95% confidence. Gao *et al*.^19^ gave a 95% confidence interval for the percentage of contribution of sexual spread in the basic reproduction number of $[0.123,45.73]$.

### Sensitivity analysis

As currently no vaccine against Zika is available, the most important control measures to decrease the transmission of Zika include decreasing mosquito bites in areas affected by the disease using insect repellents, clothes covering much of the body, mosquito nets, decreasing mosquito birth rate by getting rid of standing water where mosquitoes reproduce, mosquito killing, as well as use of condoms to prevent sexual transmission.

**Figure 9:**
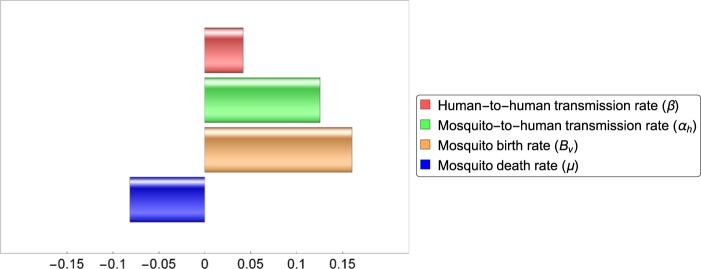
Partial rank correlation coefficients of the four parameters which can be subject to control measures. Parameters with positive PRCC are positively correlated with the total number of cases. Parameters with negative PRCC are negatively correlated with the number of infections.

Figure 9 shows the comparison of the resulting PRCC values obtained for the parameters. The results suggest that the most important factors in Zika transmission are the birth rate of mosquitoes and the transmission rate from mosquitoes to humans. Although spread through sexual contacts has a smaller effect on the number of Zika cases, it is shown to be an important factor in the transmission of Zika virus. Considering this and the results presented in the previous subsections, our study suggests that the practice of safe sex among those who have possibly contracted the disease, can significantly reduce the number of Zika cases. However, the most important ways to reduce transmission are mosquito control and protection against mosquito bites.

## Discussion

The Zika fever outbreak in South America, started in 2015, has been one of the most alarming epidemics in recent years due to its connection with microcephaly. Apart from vectorial transmission, the disease has been proved to be spread sexually, which is a previously unexperienced phenomenon among mosquito-borne diseases. Several mathematical models have been created to include the novel features of the disease, though, the majority of these did not consider the changes of mosquito populations due to periodicity of weather circumstances. In this paper, we have established a compartmental model to study the transmission of Zika virus disease including spread through sexual contacts, asymptomatic carriers and the periodicity of weather. Up to our knowledge, our model is the first compartmental model for Zika fever transmission which, besides considering both mosquito-borne and sexual transmission, the role of asymptomatic carriers and the prolonged period of sexual transmissibility, also takes into account the seasonality of weather. We fitted the model to the number of Zika cases in two countries where the Zika epidemic had a different outcome: Suriname, where there was only one peak of the epidemic and Costa Rica where two major peaks occurred in two subsequent years to show that the annual change of weather can be responsible for a recurrence of the epidemic after the autumn/winter season, though, depending on the circumstances characteristic for the given country, it is also possible that, even with the return of the rainy season, the depletion of the susceptible class might prevent a second outbreak.

Using the fittings obtained, we studied the effects of sexual transmission, a novel phenomenon not experienced before in mosquito-borne diseases. We gave an estimate for the difference in the number of cases in men and women, possibly due to the asymmetric sexual transmission rates supporting earlier statements give e.g. in^45–47^. This is especially important because of the severe side-effects in newborns of women infected during their pregnancy. Our results suggests that a 39–65% surplus in Zika cases in women in comparison with men, which can be attributed to the asymmetric transmission rates. These values are smaller than the ones given in^45–47^, however, as also suggested in those studies, this might also be attributed to the fact that women suspected to have Zika are more likely to visit their doctors. Further, we estimated the increase in the number of cases due to sexual transmission. We found that sexual transmission could be responsible for about 32–54% of the Zika infections. Our findings are in accordance with similar earlier estimates given in^19,43^. These results of us suggest that the practice of safe sex among those who have possibly been infected can significantly reduce the number of Zika cases.

Both the basic reproduction number of the time-periodic model (serving as a threshold parameter for the persistence of the epidemic) and the instantaneous reproduction number were calculated. The results are in accordance with the extinction of the epidemics in the countries of South America after one or possibly more peaks, and also with earlier results given in^19,42^.

We carried out sensitivity analysis to compare the effect of different model parameters on the number of cases. We found that mosquito birth and death rates are the most important factors in the spread of Zika, but sexual transmission rate has also a significant effect on the prevalence of the disease, supporting the earlier statements about the possibility of reduction of the number of Zika cases by decreasing the probability of sexual transmission.

## Supplementary information

Supplementary Information

## Data Availability

The datasets generated during and/or analysed during the current study are available from the corresponding author on reasonable request.
