# Supplementary material for: Impact of weather seasonality and sexual transmission on the spread of Zika fever
Source: Sci Rep. 2019 Nov 19;9:17055. doi: 10.1038/s41598-019-53062-z (PMC6863851; doi:10.1038/s41598-019-53062-z)
Supplement: Supplementary file 1 — Supplementary Information [file 41598_2019_53062_MOESM1_ESM.pdf]

Impact of weather seasonality and sexual transmission on the spread of Zika fever

Attila Dénes*[1], Mahmoud A. Ibrahim[1,2], Lillian Oluoch[1], Miklós Tekeli[1], and Tamás Tekeli[1]

[1]Bolyai Institute, University of Szeged,
Aradi vértanúk tere 1., Szeged H-6720, Hungary
[2]Department of Mathematics, Faculty of Science, Mansoura University,
Mansoura 35516, Egypt

Supplementary information

S.1 The governing equations

In accordance with the transmission diagram in Figure 3 and the parameter description given in Table 1, introducing the notation $N_h(t)$ and $N_f(t)$, for the total human and total female human population, respectively, the corresponding system of differential equations takes the form

$$\begin{aligned}
S_f'(t) &= \tfrac{B_h}{2} - \beta \tfrac{\kappa_e E_m(t) + \kappa_a I_m^a(t) + I_m^s(t) + \kappa_r I_m^r(t)}{N_f(t)} S_f(t) - \tfrac{\tilde{\alpha}_h(t) I_v(t)}{N_h(t)} S_f(t) - d S_f(t), \\
E_f'(t) &= \beta \tfrac{\kappa_e E_m(t) + \kappa_a I_m^a(t) + I_m^s(t) + \kappa_r I_m^r(t)}{N_f(t)} S_f(t) + \tfrac{\tilde{\alpha}_h(t) I_v(t)}{N_h(t)} S_f(t) - \nu_h E_f(t) - d E_f(t), \\
I_f^{a\prime}(t) &= \theta \nu_h E_f(t) - \gamma_a I_f^a(t) - d I_f^a(t), \\
I_f^{s\prime}(t) &= (1-\theta) \nu_h E_f(t) - \gamma_s I_f^s(t) - d I_f^s(t), \\
R_f'(t) &= \gamma_a I_f^a(t) + \gamma_s I_f^s(t) - d R_f(t), \\
S_m'(t) &= \tfrac{B_h}{2} - \tfrac{\tilde{\alpha}_h(t) I_v(t)}{N_h(t)} S_m(t) - d S_m(t), \\
E_m'(t) &= \tfrac{\tilde{\alpha}_h(t) I_v(t)}{N_h(t)} S_m(t) - \nu_h E_m(t) - d E_m(t), \\
I_m^{a\prime}(t) &= \theta \nu_h E_m(t) - \gamma_a I_m^a(t) - d I_m^a(t), \\
I_m^{s\prime}(t) &= (1-\theta) \nu_h E_m(t) - \gamma_s I_m^s(t) - d I_m^s(t), \\
I_m^{r\prime}(t) &= \gamma_a I_m^a(t) + \gamma_s I_m^s(t) - \gamma_r I_m^r(t) - d I_m^r(t), \\
R_m'(t) &= \gamma_r I_m^r(t) - d R_m(t), \\
S_v'(t) &= \tilde{B}_v(t) - \tilde{\alpha}_v(t) \tfrac{\eta_e E_f(t) + \eta_a I_f^a(t) + I_f^s(t) + \eta_e E_m(t) + \eta_a I_m^a(t) + I_m^s(t) + I_m^r(t)}{N_h(t)} S_v(t) - \mu S_v(t), \\
E_v'(t) &= \tilde{\alpha}_v(t) \tfrac{\eta_e E_f(t) + \eta_a I_f^a(t) + I_f^s(t) + \eta_e E_m(t) + \eta_a I_m^a(t) + I_m^s(t) + I_m^r(t)}{N_h(t)} S_v(t) - (\nu_v + \mu) E_v(t), \\
I_v'(t) &= \nu_v E_v(t) - \mu I_v(t).
\end{aligned} \tag{S.1}$$

*Corresponding author. Email: denesa@math.u-szeged.hu

Here, B_h and d denote human birth and death rate, respectively, β stands for transmission rate from symptomatically infected men to susceptible women, while for transmission rates from exposed, asymptotically infected and convalescent men to women are obtained by multiplying β by κ_e, κ_a and κ_r, respectively. The parameter θ is the fraction of asymptomatically infected among all infected people. The length of latent period for humans is $1/\nu_h$ and $1/\gamma_a, 1/\gamma_s$ denote the length of infected period for asymptotically and symptomatically infected people, respectively, while $1/\gamma_r$ is the length of the period during which recovered men are still infectious through sexual contact. The functions $\tilde{\alpha}_h(t)$, $\tilde{\alpha}_v(t)$ and $\tilde{B}_v(t)$ denote transmission rate from an infectious mosquito to a susceptible human, transmission rate from an infected human to a susceptible mosquito and mosquito birth rate, respectively. These functions are assumed to be time periodic with one year as a period and, following e.g. [49] they are assumed to be of the form $\tilde{\alpha}_h(t) = \alpha_h \cdot \left(\sin\left(\frac{2\pi}{P}t + b\right) + a\right)$, $\tilde{\alpha}_v(t) = \alpha_v \cdot \left(\sin\left(\frac{2\pi}{P}t + b\right) + a\right)$ and $\tilde{B}_v(t) = B_v \cdot \left(\sin\left(\frac{2\pi}{P}t + b\right) + a\right)$, respectively, where P is period length, a, b are free adjustment parameters and α_h, α_v, B_v are constants. Just like in the case of human-to-human transmission, we also introduce the modification parameters η_e, η_a for infectiousness of exposed and asymptomatically infected people, respectively. We have $1/\nu_v$ for the length of the latent period for mosquitoes, while average life span of mosquitoes is given by $1/\mu$.

The introduction of a nonautonomous model was needed to reproduce an epidemic with multiple peaks; this is supported by Figure S.1 where we tried to fit the autonomous model obtained from (S.1) by setting the time-dependent parameters (mosquito birth and death rates and biting rates) constant.

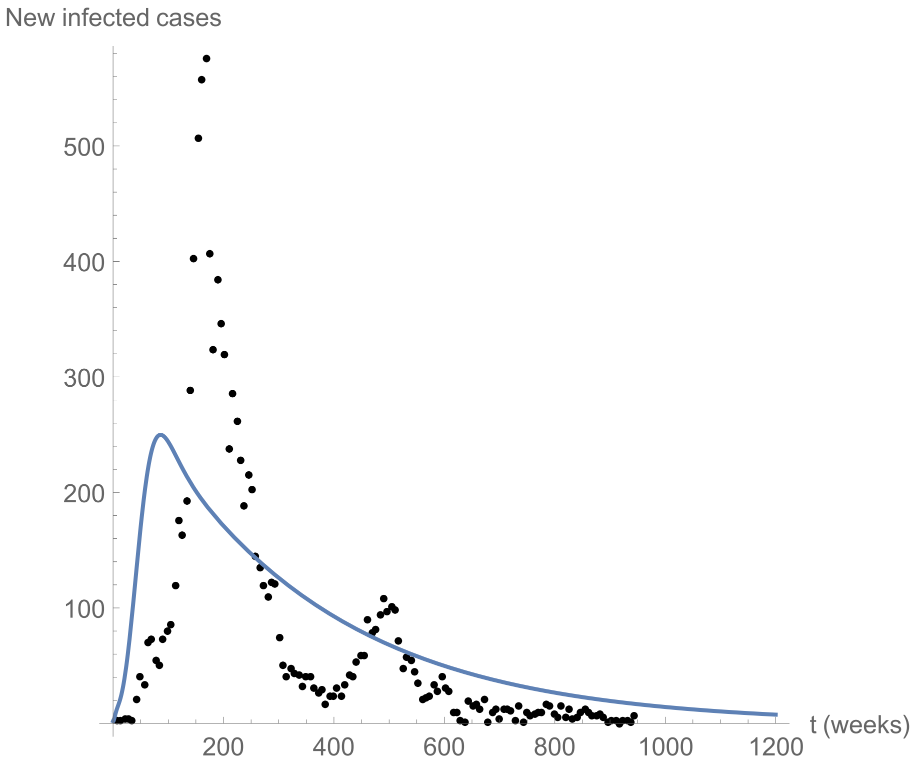

Figure S.1: Best fitting solution of the autonomous model for the case of Costa Rica.

S.2 Derivation of the basic reproduction number of the autonomous model

To calculate the basic reproduction number $\mathcal{R}_0$ of the autonomous model obtained from (S.1) by setting the time-dependent parameters (mosquito birth and death rates and biting rates) constant, i.e. $\tilde{B}_v(t) \equiv B_v$, $\tilde{\alpha}_h(t) \equiv \alpha_h$ and $\tilde{\alpha}_v(t) \equiv \alpha_v$, we follow the general approach established in [50]. Given the infectious states E_f, I_f^a, I_f^s, E_m, I_m^a, I_m^s, I_m^r, E_v and I_v in (S.1), we can create the transmission vector $\mathcal{F}$ representing the new infections flowing only into the exposed compartments given by

$$\mathcal{F} = \Bigg(\beta\frac{\kappa_e E_m + \kappa_a I_m^a + I_m^s + \kappa_r I_m^r}{N_f} S_f + \frac{\alpha_H I_V}{N_H} S_F, 0, 0, \frac{\alpha_h I_v}{N_h} S_m, 0, 0, 0, \frac{\alpha_h I_v}{N_h} S_m,$$
$$0, 0, \alpha_v \frac{\eta_e E_f + \eta_a I_f^a + I_f^s + \eta_e E_m + \eta_a I_m^a + I_m^s + I_m^r}{N_h} S_v, 0\Bigg)^{\mathsf{T}},$$

while the transition vector $\mathcal{V}$ which denotes the outflow from the infectious compartments in (S.1), is given by

$$\mathcal{V} = \Big((\nu_h + d)E_f, (\gamma_a + d)I_f^a - \theta\nu_h E_f, (\gamma_s + d)I_f^s - (1-\theta)\nu_h E_f, (\nu_h + d)E_m, (\gamma_a + d)I_m^a - \theta\nu_h E_m,$$
$$(\gamma_s + d)I_m^s - (1-\theta)\nu_h E_m, (\gamma_r + d)I_m^r - \gamma_a I_m^a - \gamma_s I_m^s, (\nu_v + \mu)E_v, \mu I_v - \nu_v E_v\Big)^{\mathsf{T}}$$

here we note that the notations α_h, α_v and B_v stand for the (now constant) transmission rate from an infectious mosquito to a susceptible human, transmission rate from an infected human to a susceptible mosquito and mosquito birth rate, respectively. Substituting the values in the disease-free equilibrium $N_h = \frac{B_h}{d}$ and $N_f = \frac{B_h}{2d}$, we compute the Jacobian F from $\mathcal{F}$ given by

$$F = \begin{pmatrix}
0 & 0 & 0 & \beta\kappa_e & \beta\kappa_a & \beta & \beta\kappa_r & 0 & \frac{\alpha_h}{2} \\
0 & 0 & 0 & 0 & 0 & 0 & 0 & 0 & 0 \\
0 & 0 & 0 & 0 & 0 & 0 & 0 & 0 & 0 \\
0 & 0 & 0 & 0 & 0 & 0 & 0 & 0 & \frac{\alpha_h}{2} \\
0 & 0 & 0 & 0 & 0 & 0 & 0 & 0 & 0 \\
0 & 0 & 0 & 0 & 0 & 0 & 0 & 0 & 0 \\
0 & 0 & 0 & 0 & 0 & 0 & 0 & 0 & 0 \\
\frac{\alpha_v B_v \eta_e d}{\mu B_h} & \frac{\alpha_v B_v \eta_a d}{\mu B_h} & \frac{\alpha_v B_v d}{\mu B_h} & \frac{\alpha_v B_v \eta_e d}{\mu B_h} & \frac{\alpha_v B_v \eta_a d}{\mu B_h} & \frac{\alpha_v B_v d}{\mu B_h} & \frac{\alpha_v B_v d}{\mu B_h} & 0 & 0 \\
0 & 0 & 0 & 0 & 0 & 0 & 0 & 0 & 0
\end{pmatrix}$$

and the Jacobian V from $\mathcal{V}$ given by

$$V = \begin{pmatrix}
d+\nu_h & 0 & 0 & 0 & 0 & 0 & 0 & 0 & 0 \\
-\theta\nu_h & \gamma_a+d & 0 & 0 & 0 & 0 & 0 & 0 & 0 \\
-(1-\theta)\nu_h & 0 & \gamma_s+d & 0 & 0 & 0 & 0 & 0 & 0 \\
0 & 0 & 0 & d+\nu_h & 0 & 0 & 0 & 0 & 0 \\
0 & 0 & 0 & -\theta\nu_h & \gamma_a+d & 0 & 0 & 0 & 0 \\
0 & 0 & 0 & -(1-\theta)\nu_h & 0 & \gamma_s+d & 0 & 0 & 0 \\
0 & 0 & 0 & 0 & -\gamma_a & -\gamma_s & \gamma_r+d & 0 & 0 \\
0 & 0 & 0 & 0 & 0 & 0 & 0 & \mu+\nu_v & 0 \\
0 & 0 & 0 & 0 & 0 & 0 & 0 & -\nu_v & \mu
\end{pmatrix},$$

therefore the characteristic polynomial of the next generation matrix FV^{-1} is

$$\lambda^6 \left(R_{mf} R_{vh} R_{fv} + R_{vh}(R_{fv} + R_{mv})\lambda - \lambda^3\right) = 0$$

where

$$
\begin{aligned}
R_{mf} &= \tfrac{\beta\kappa_e}{(d+\nu_h)} + \tfrac{\beta(1-\theta)\nu_h}{(\gamma_s+d)(d+\nu_h)} + \tfrac{\beta\kappa_a\theta\nu_h}{(\gamma_a+d)(d+\nu_h)} + \tfrac{\beta\kappa_r\nu_h(\gamma_a\gamma_s+\theta\gamma_a d+(1-\theta)\gamma_s d)}{(\gamma_a+d)(\gamma_r+d)(\gamma_s+d)(d+\nu_h)} \\
R_{vh} &= R_{vm} = R_{vf} = \tfrac{\alpha_h\nu_v}{2\mu(\mu+\nu_v)} \\
R_{fv} &= \tfrac{\alpha_v B_v\eta_e d}{\mu B_h(d+\nu_h)} + \tfrac{\alpha_v B_v\eta_a\theta d\nu_h}{\mu B_h(\gamma_a+d)(d+\nu_h)} + \tfrac{\alpha_v B_v(1-\theta)d\nu_h}{\mu B_h(\gamma_s+d)(d+\nu_h)} \\
R_{mv} &= \tfrac{\alpha_v B_v\eta_e d}{\mu B_h(d+\nu_h)} + \tfrac{\alpha_v B_v(1-\theta)d\nu_h}{\mu B_h(\gamma_s+d)(d+\nu_h)} + \tfrac{\alpha_v B_v\eta_a\theta d\nu_h}{\mu B_h(\gamma_a+d)(\gamma_s+d)(d+\nu_h)} + \tfrac{\alpha_v B_v d\nu_h(\gamma_a\gamma_s+\theta\gamma_a d+(1-\theta)\gamma_s d)}{\mu B_h(\gamma_a+d)(\gamma_r+d)(\gamma_s+d)(d+\nu_h)}
\end{aligned}
$$

The characteristic polynomial therefore is the following cubic equation given by

$$\lambda^3 - R_{vh}(R_{fv}+R_{mv})\lambda - R_{mf}R_{vh}R_{fv} = 0$$

According to [50], the basic reproduction number $\mathcal{R}_0$ is the spectral radius of FV^{-1}. Thus, the basic reproduction number $\mathcal{R}_0$ corresponds to the dominant eigenvalue given by the root of the cubic equation

$$
\begin{aligned}
\mathcal{R}_0 = &\frac{\sqrt[3]{2}R_{vh}(R_{fv}+R_{mv})}{\sqrt[3]{27R_{mf}R_{vh}R_{fv}+\sqrt{729R_{mf}^2R_{vh}^2R_{fv}^2-108R_{vh}^3(R_{fv}+R_{mv})^3}}} \\
&+\frac{\sqrt[3]{27R_{mf}R_{vh}R_{fv}+\sqrt{729R_{mf}^2R_{vh}^2R_{fv}^2-108R_{vh}^3(R_{fv}+R_{mv})^3}}}{3\sqrt[3]{2}}
\end{aligned}
$$

S.3 Numerical estimation of the basic reproduction number of the nonautonomous model

In periodic epidemiological models, one can determine the basic reproduction as the spectral radius of a linear integral operator on a space of periodic functions (see [39] for details). The value of the basic reproduction number generally cannot be calculated analytically, but one can numerically approximate it. To do so, first, one writes the model

$$x'(t) = f(x(t))$$

as

$$x_i' = \mathcal{F}_i(t,x) - \mathcal{V}_i(t,x), \qquad i = 1,\dots,n,$$

where $\mathcal{F}_i(t,x)$ stands for the be the rate of new infections in compartment i and $\mathcal{V}_i(t,x) = \mathcal{V}_i^-(t,x) - \mathcal{V}_i^+(t,x)$ with $\mathcal{V}_i^+(t,x)$ denoting the rate of transfer into compartment i by all means different from new infections, and $\mathcal{V}_i^-(t,x)$ is the rate of transfer out of the ith compartment. Let m be the number of infectious compartments. The matrices $F(t)$ and $V(t)$ are defined as

$$F(t) = \left(\tfrac{\partial\mathcal{F}_i(t,x_0(t))}{\partial x_j}\right)_{1\le i,j\le m} \quad \text{and} \quad V(t) = \left(\tfrac{\partial\mathcal{V}_i(t,x_0(t))}{\partial x_j}\right)_{1\le i,j\le m}$$

and consider the linear periodic equation

$$\frac{\mathrm{d}w}{\mathrm{d}t} = \left(-V(t) + \tfrac{F(t)}{\lambda}\right)w, \qquad t\in\mathbb{R} \tag{S.2}$$

with $\lambda \in (0,\infty)$. First we find the monodromy matrix Φ of (S.2) by finding m linearly independent solutions, most simply by taking the (linearly independent) unit vectors of $\mathbb{R}^m$ as initial values.

Then we select an initial guess λ_0 and determine the spectral radius $\rho(\Phi(\lambda_0))$. If this ρ_0 is less than 1, then we set $\lambda_- = \lambda_0$ and increase our guess λ_0 to find an upper bound λ_+ with which $\rho(\Phi(\lambda_+))$ is larger than 1. If $\rho_0 > 1$, we proceed similarly, but the other way around. Then we keep choosing $\lambda_j \in (\lambda_-, \lambda_+)$ e.g. as $(\lambda_- + \lambda_+)/2$ and if $\rho(\Phi(\lambda_j)) < 1$, we set $\lambda_- = \lambda_j$, otherwise $\lambda_+ = \lambda_j$. We proceed until $\lambda_+ - \lambda_- < \varepsilon$ for some sufficiently small ε. For more details see e.g. [40].
